# Anti-Inflammatory and Antioxidant Effects of Regular Consumption of Cooked Ham Enriched with Dietary Phenolics in Diet-Induced Obese Mice

**DOI:** 10.3390/antiox9070639

**Published:** 2020-07-21

**Authors:** Antonio Serrano, Antonio González-Sarrías, Francisco A. Tomás-Barberán, Antonio Avellaneda, Amadeo Gironés-Vilaplana, Gema Nieto, Gaspar Ros-Berruezo

**Affiliations:** 1 Department of Food Technology, Nutrition and Food Science, Veterinary Faculty University of Murcia, Campus de Espinardo, Espinardo, 30100 Murcia, Spain; antonio.serrano5@um.es (A.S.); gros@um.es (G.R.-B.); 2 Cátedra de Seguridad y Sostenibilidad Alimentaria Grupo Fuertes-Universidad de Murcia, 30003 Murcia, Spain; 3 Laboratory of Food & Health, Research Group on Quality, Safety, and Bioactivity of Plant Foods, CEBAS-CSIC, Campus de Espinardo, 30100 Murcia, Spain; agsarrias@cebas.csic.es (A.G.-S.); fatomas@cebas.csic.es (F.A.T.-B.); 4 R&D Department, ElPozo Alimentación S.A., Alhama de Murcia, 30840 Murcia, Spain; antonio.avellanedagoicuria@elpozo.com (A.A.); amadeo.girones@elpozo.com (A.G.-V.)

**Keywords:** ham, oxidative stress, inflammatory biomarkers, polyphenols

## Abstract

Oxidative damage and chronic inflammation have been proven as one of the major factors associated with obesity, which increases the incidence of non-communicable chronic diseases. In this sense, the development of new functional products aiming at the palliation of oxidative stress and inflammatory disruption can be a determining factor for public health as seen in previous researches. In this study, a blend of potentially bioavailable dietary phenolics was added to low sodium and low-fat cooked ham. A diet-induced obesity model in C57/BL6J mice has been used for testing the effectiveness of the phenolic blend and the new functionalized product, which bioavailability was tested by UPLC-ESI-QTOF-MS. After obesity induction, different oxidative and inflammatory biomarkers were evaluated. Results in the murine induced obesity model, demonstrate a robust statistically significant improvement in key parameters related with obesity risk in the groups feed with a phenolic-enriched diets (P) + high-fat diet (HFD) and phenolic enriched cooked ham (PECH) + HFD. In both groups there was an improvement in body composition parameters, inflammatory biomarkers and antioxidant enzymes levels. Specifically in the group feed with the phenolic enriched cooked ham (PECH + HFD) there was an improvement of total fat volume (23.08% reduction), spleen index (22.04% of reduction), plasmatic MCP-1 (18% reduction), IL-6 (38.94% reduction), IL-10 (13.28% reduction), TNF-α (21.32% reduction), gut IL-1β (10.86% reduction), gut IL-6 (13.63% reduction) and GPx (60.15% increase) and catalase (91.37% increase) enzymes. Thus, the functionalized ham could be considered an appropriate dietary polyphenol source, which might improve the oxidative and inflammatory status and could finally result in the potential decrease of the risk of certain non-communicable chronic diseases.

## 1. Introduction

Obesity has become the most significant public health problem [1], and it is considered as the result of excessive energy intake compared to the energy expenditure [2]. It characterized by a body mass index equal or higher than 30 kg/m^2^, with an increase in adipocyte number and size [3]. Obesity leads to augmented cardiovascular and diabetes risk factors [4] with some disorders such as dyslipidaemia, non-alcoholic fatty liver disease, as well as oxidative and inflammatory disruptions [5,6,7,8]. Therefore, the reversal of these disorders and the prevention of obesity are essential challenges for society.

Nowadays, obesity is usually treated with changes in lifestyle [9] or drugs [10]. Recent studies have demonstrated that dietary polyphenols can contribute to improve obesity disorders due to their antioxidant and anti-inflammatory properties [11,12,13]. (Poly)phenolic compounds are the most common and ubiquitous groups of secondary metabolites, widely distributed in plant foods such as fruits, vegetables and beverages. They have been reported to exhibit a broad spectrum of potential biological activities, including their antioxidant and anti-inflammatory properties, related to the prevention of chronic diseases such as cancer, diabetes, cardiovascular, and neurodegenerative diseases [14,15]. For decades, their potential anti-inflammatory effects have been associated with their antioxidant activity reduce reactive oxygen species (ROS) and nitric oxide (NO) production, reduced lipid peroxidation), and by the modulation of a plethora of cell-signalling pathways and inflammation mediators (cytokines, eicosanoids, etc.) [16]. However, despite the numerous preclinical studies, the clinical evidence for these health benefits is still elusive, at least in part, as their health effects depend on both, their bioavailability and metabolism, and the interindividual response after their intake [17]. Even so, although only a small number of dietary phenolics are considered bioavailable, their potential benefits may be achieved by the consumption of a natural phenolic-enriched diet, as well as by their administration as food supplements or nutraceuticals. In this sense, in recent years, to respond to consumer’s demands, functionalizing food through phenolic enrichment has been considered an advantage for dealing with chronic diseases, including obesity disorders.

Meat and meat products are widely consumed in most dietary patterns [18,19]. They are a source of nutrients such as high-quality proteins, vitamins (B6 and B12) and minerals (Fe, Zn or Se among others). Regarding lipid profile, due to animal genetic, nutrition and managements, there is a wide range of lean meats (less than 5% of fat, and a reduced saturated fatty acid profile), that are included in some weight-loss diets [20]. There is an industrial trend drive by consumers demand to improve the nutritional profile of cooked hams by lowering its lipid content (less than 1.5%), reduce saturated fatty acids and maintain its high protein content (18,5%) and quality. As previously mentioned, plant polyphenols provide clear health benefits. The use of polyphenols in the meat industry have been explore aiming to improve the preservation of meat [21,22] and transform meat products in a source of polyphenols to produce a beneficial effect on the health of consumers [23,24]. One of the most consumed pieces of lean meat is cooked ham, which consists of brined hind legs of swine, either smoked or not [25].

The present study aims to demonstrate that the regular consumption of a meat product enriched with plant polyphenols have a positive impact in a murine model, mainly on oxidative stress, chronic inflammation, adiposity and obesity. For this purpose, the effects of a ham with improved nutritional profile (low in salt, fat and saturated fatty acids) incorporating a mixture of polyphenols (catechins, chlorogenic acids and hydroxytyrosol—HXT—selected for their efficacy on obesity disorders [11,26,27,28]) was investigated in a murine model.

## 2. Materials and Methods 

### 2.1. Ham Preparation and Diet Composition

Cooked ham used for this study was provided by a local meat industry (ElPozo Alimentación, S.A., Alhama de Murcia, Murcia, Spain). This type of cooked ham has an improved nutritional profile with a total fat content below 1.5%, and an amount of saturated fat and sodium below 0.5% (BienStar^®^, ElPozo Alimentación, Alhama de Murcia, Murcia, Spain). It was used as the base product for developing the new phenolic-enriched ham. Uncooked ham dough was mixed 5% *w/w* with water for the control sample and with a natural antioxidant aqueous solution of 12.5 mg/mL of chlorogenic acid, 10 mg/mL of catechins and 1.5 mg/mL of HXT. Both hams were cooked at 80 °C for 40 min. After being cooked, hams were lyophilized (Telstar Lyoquest Lyophilizer, Tarrasa, Barcelona, Spain) to obtain a powder suitable for mixing with the mice diet.

Five diets were prepared for the present study: (1) Pelletized standard diet (SD) (Teklad Global 14% Protein Rodent Maintenance Diet. Envigo, Barcelona, Spain) and (2) high-fat diet (HFD) (Teklad Custom Diet TD.06414 60/fat. Envigo) were obtained from the manufacturer. Then, (3) phenolic-enriched high-fat diet (P + HFD) was obtained by adding the natural antioxidant mix previously described to a final concentration of 166 mg/kg to high-fat diet composition, (4) lyophilized cooked ham enriched high-fat diet (CH + HFD) was obtained by adding the lyophilized standard cooked ham to a final concentration of 50 g/kg, and (5) lyophilized phenolic enriched cooked ham enriched high-fat diet (PECH + HFD) was obtained by adding the lyophilized phenolic-enriched cooked ham to a final concentration of 50 g/kg. 

Casein, lard, and maltodextrin, which are the primary macronutrient sources of HFD, were used for standardizing macronutrient profile of every diet in the intervention phase after adding or not both lyophilized cooked hams (Table 1). All HFD diets were re-pelletized.

### 2.2. Animals, Treatments and Ethical Considerations

The experiment was approved by the Animal Experimentation Ethics Committee of the University of Murcia, Spain, following the directive 2010/63/EU of the European Parliament and of the Council on the protection of animals used for scientific purposes.

For the present study, 36 male C57/BL6J mice six weeks old were provided by Envigo. They were randomly housed in nine cages with four animals each. They were divided based on the previously described diet interventions. Mice were fed *ad libitum* with the SD during the adaptation period (weeks 6 to 11). SD was maintained for control mice, housed in cage 1, and obesity was induced through the high-fat diet along five weeks (weeks 11 to 16), cages 2 to 9. Once the diet-induced obesity (DIO) period was over, HFD was maintained for obesity control cages (2 and 3), and the following intervention diets were applied: P+HFD to cages 4–5, CH + HFD to cages 6–7 and PECH + HFD to cages 8–9. Consumed food was quantified to detect possible differences throughout the study.

### 2.3. Sampling

At the end of treatments, blood was collected from the submandibular vein into a heparin-coated tube. Blood samples were centrifuged at 4000 g for 10 min (4 °C), and plasma was collected and frozen at −80 °C for further analysis. Mice were housed by groups of 4 individuals from the same treatment in metabolic cages with a separation system for feces and urine, and both were collected. 

For protein (cytokine) determination, gut samples were obtained using Tissue Ruptor and Qproteome Mammalian Protein Prep Kit (Qiagen, Hilden, Germany) following the manufacturer’s instructions.

Urine samples were vortexed during a minute, centrifuged at 300× *g* for 10 min at 4 °C. Next, urine samples were diluted 1:5 with acidified water (0.1% formic acid), filtered through a 0.22 µm polyvinylidene fluoride (PVDF) filter and injected in the UPLC-ESI-QTOF-MS equipment.

Livers were collected in 10% phosphate-buffered formalin and stored less than 24 h at 4 °C. Sections of 5 µm were obtained using Leica Microtome RM2155 (Leica, Wetzlar, Germany) stained with hematoxylin and eosin. Images were obtained using a digital camera Leica DC500 with Leica DMRB optical microscope. Samples were analysed in triplicate for fat content according to AOAC methods [29]

### 2.4. Body and Spleen Weight and Body Composition

Mice were weighed weekly, between 4:00−5:00 p.m., using a precision weight scale (0.1 g) (Highland Adam 600 × 0.01 g, UK) and right after the sacrifice. The spleen was immediately removed after collecting blood samples and sacrificing and then weighted. The following formula was used for obtaining the spleen index: Spleen index (mg/g) = spleen weight (mg)/ live body weight before sacrifice (g).

**Figure 1:**
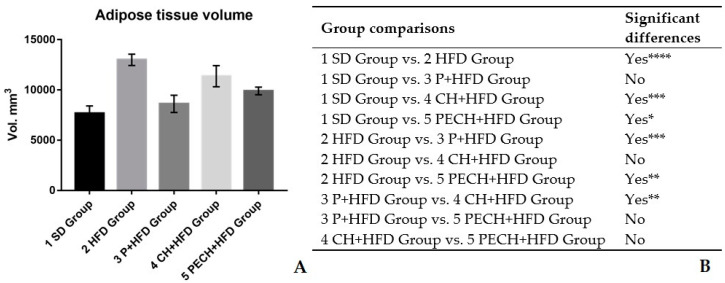

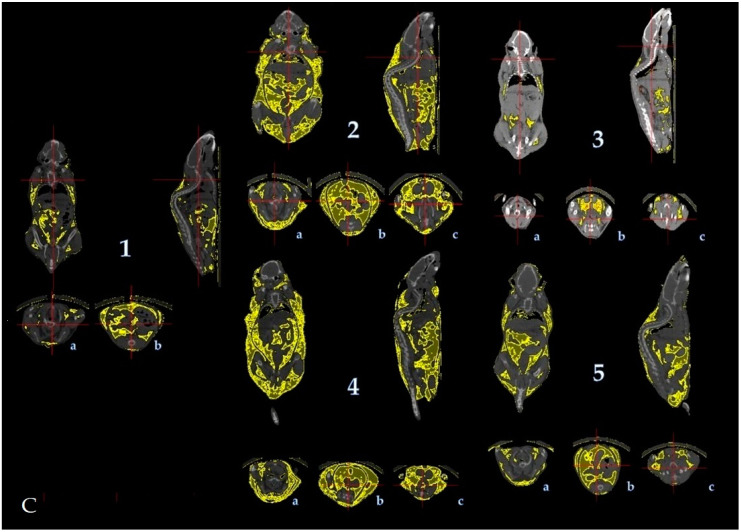
Effect of the different diets on the adipose tissue volume. (**A**) Adipose tissue volume expressed as mm^3^ at the end of the study. Numbers and groups correspond as following: (1) Standard diet, (SD), (2) high-fat diet (HFD), (3) phenolic-enriched high-fat diet (P+HFD), (4) lyophilized cooked ham enriched high-fat diet (CH+HFD) and (5) lyophilized phenolic enriched cooked ham enriched high-fat diet (PECH). (**B**) Statistical comparison Significant differences are expressed as * *p* < 0.05 ** *p* < 0.01 *** *p* < 0.001 **** *p* < 0.0001 (**C**) PET/SPECT/CT computerized images showing areas with a density attributed to adipose tissue coloured by yellow (voxels).

Total fat volume was determined, immediately after mice euthanasia, through the method described by Moreno [30] using the Albira CT system -a small animal positron emission tomography (PET)/single photon emission computed tomography (SPECT)/ computed tomography (CT) imaging system- (Bruker Molecular Imaging, Woodbridge, CT, USA). Images were computerized (using the filtered back-projection algorithm via the Albira Suite 5.0 Reconstructor), 3D image obtained (Figure 1) had 125-µm isotropic voxels (each of an array of elements of volume that constitute a notional three-dimensional space, especially each of an array of discrete elements into which a representation of a three-dimensional object is divided) and was analysed through PMOD software (PMOD Technologies, Zurich, Switzerland). Finally, images were segmented according to adipose tissue density and voxels corresponding to adipose tissue were interpreted as total fat volume, expressed as mm^3^.

### 2.5. Evaluation of Antioxidant Capacity

The evaluation of antioxidant capacity and antioxidant enzymes in plasma were performed following the methods described in [31] Glutathione peroxidase (GPx) assay was performed based on the method described by Paglia and Valentine [32] with a commercially available kit (Ransel test kit, Randox Laboratories Ltd., Crumlin, County Antrim, UK). Catalase activity was determined according to Johansson and Borg [33]. Ferric reducing antioxidant power (FRAP) assay was performed according to Benzie and Strain [34], and paraoxonase 1 (PON1) was measured following the method described by Tvarijonaviciute et al. [35].

### 2.6. Cytokine Measurement

The cytokines monocyte chemoattractant protein 1 (MCP-1), interleukin (IL)-1β, IL-6, IL-10, and tumoral necrosis factor-alpha (TNF-α) were measured using Luminex multiplex immunoassay technology. A Luminex MAGPIX system with a MCYTOMAG-70K plate (Merck KGaA, Darmstadt, Germany) was used. According to the manufacturer instructions, a total of 25 μL of plasma or gut lysate was used to determine cytokine concentrations in triplicate using the Luminex xPONENT ^®^ software (Hertogenbosch, The Netherlands). The results were obtained in pg/mL.

### 2.7. OxLDL Measurement

Plasma oxidized low-density lipoprotein (OxLDL) concentrations were determined using commercially available standard ELISA kits (Mouse Oxidized Low-Density Lipoprotein (OxLDL) ELISA Kit 96-strip-wells, mybiosource.com, San Diego, CA, USA) following the manufacturer’s instructions. All samples were analysed at least in duplicate.

### 2.8. UPLC-ESI-QTOF-MS Analysis of Urine

Phenolic-derived metabolites were identified in urine by UPLC-ESI-QTOF-MS, as previously described [36]. The injection volume was 3 µL. A target screening strategy was applied for the qualitative screening of 60 possible metabolites that could be present after consumption of the polyphenol mix. Including both parent phenolic compounds, phase II derived metabolites (glucuronides, sulphates, etc.) and phenolic acids (Table S1). Organic solvents of analytical grade were obtained from Merck KGaA and Milli-Q ultrapure water from Millipore Corp. (Bedford, MA, USA).

### 2.9. Statistics

Statistical analysis and graphics were performed using the GraphPad Prism 7.0 software for Windows (GraphPad Software, San Diego, CA, USA) and Sigma Plot 13.0 (Systat Software, San Jose, CA, USA). Multiple comparisons were performed using one-way, or two-way ANOVA followed by Tukey multiple comparison test. Results are expressed as the mean plus or minus of the standard error of the mean (SEM). The p-values of less than 0.05 were considered significant.

## 3. Results

### 3.1. Changes in Body Weight and Body Composition 

**Figure 2:**
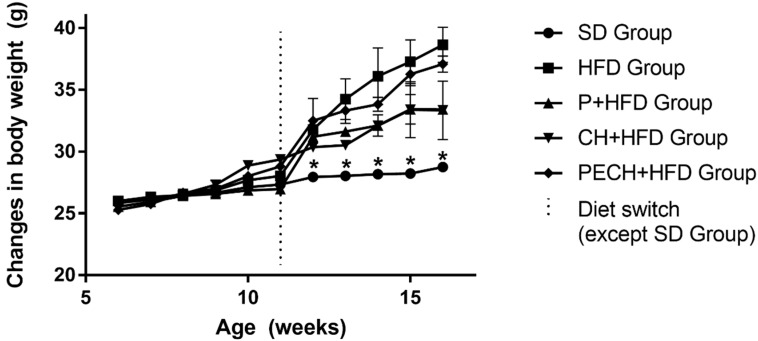
Changes in body weight during the study. Each group starts with SD (standard diet, *n* = 4) during the adaptation period. Diets were switched from SD to each high-fat diet intervention (high-fat diet (HFD, *n* = 8), phenolic-enriched high-fat diet (P + HFD, *n* = 8), lyophilized cooked ham enriched high-fat diet (CH + HFD, *n* = 8) and lyophilized phenolic enriched cooked ham enriched high-fat diet (PECH+HFD, *n* = 8) at week 11. Significant differences between SD Group and each other are expressed as * *p* < 0.05.

As shown in Figure 2, weight changes were small during the adaptation period with SD. Significant differences were found after the first week of diet-induced obesity period. Bodyweight changes comply with specification sheet from Teklad Custom Diet TD.06414 60/fat. It can be stated that enriched HFD diets have not an anorexigenic effect because average food intake among groups did not show significant differences (data not shown) and because body weight differences between HFD groups were not statistically significant.

Significant differences were observed between SD group and HFD group on total adipose volume, verifying the fattening effect of high-fat diet compared with standard diet. No differences were observed between SD and P + HFD, indicating that P+HFD diets could control adipogenesis. There were no differences between HFD and CH + HFD group. However, PECH + HFD group presented less adipose volume than HFD group. 

**Figure 3:**
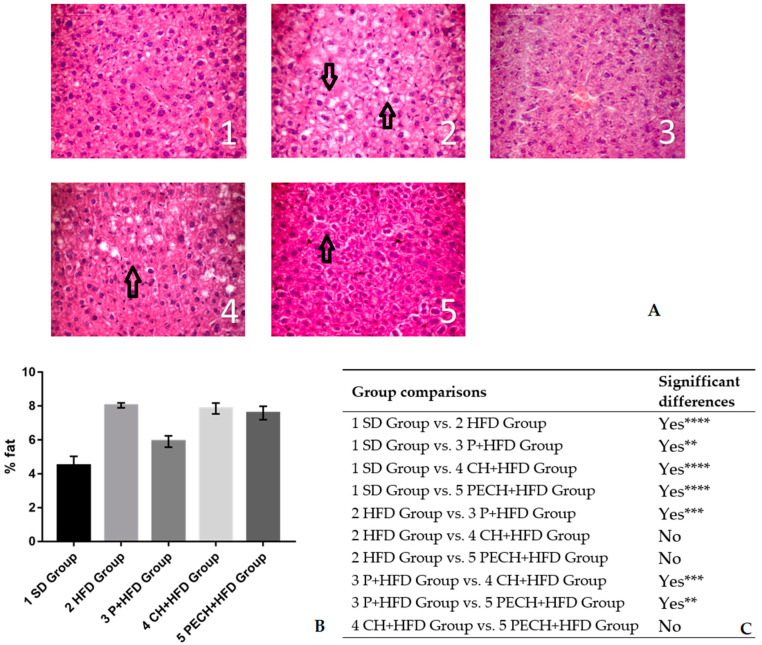
Effect of the different diets on the fat content in liver. (**A**) Liver cuts stained with hematoxylin-eosin. Non-stained areas correspond with lipid deposits between hepatocytes, pointed by black arrows. Numbers and groups correspond as following: (1) Standard diet, (SD), (2) high-fat diet (HFD), (3) phenolic-enriched high-fat diet (P + HFD), (4) lyophilized cooked ham enriched high-fat diet (CH + HFD) and (5) lyophilized phenolic enriched cooked ham enriched high-fat diet (PECH). (**B**) The graph shows total fat content in humid samples as %. (**C**) Statistical comparison. Significant differences are expressed as ** *p* < 0.01 *** *p* < 0.001 **** *p* < 0.0001.

Total fat content in liver was calculated to evaluate the effects of high fat diets over liver steatosis ans presented in Figure 3. A higher adiposity was observed in stained cuts from livers corresponding to HFD and CH + HFD diets (Figure 3A). Every obese group had higher liver fat concentration than SD group (Figure 3B). However, P + HFD group had lower concentrations of fat content than CH + HFD and PECH + HFD groups. A higher phenolic content was correlated with less liver fat content.

Changes in body weight and body composition have demonstrated the effectivity of every high fat diet inducing obesity in mice. Nevertheless, diets including phenolic compounds have shown lower obesity induction than those without phenolics.

### 3.2. Inflammatory Markers

**Figure 4:**
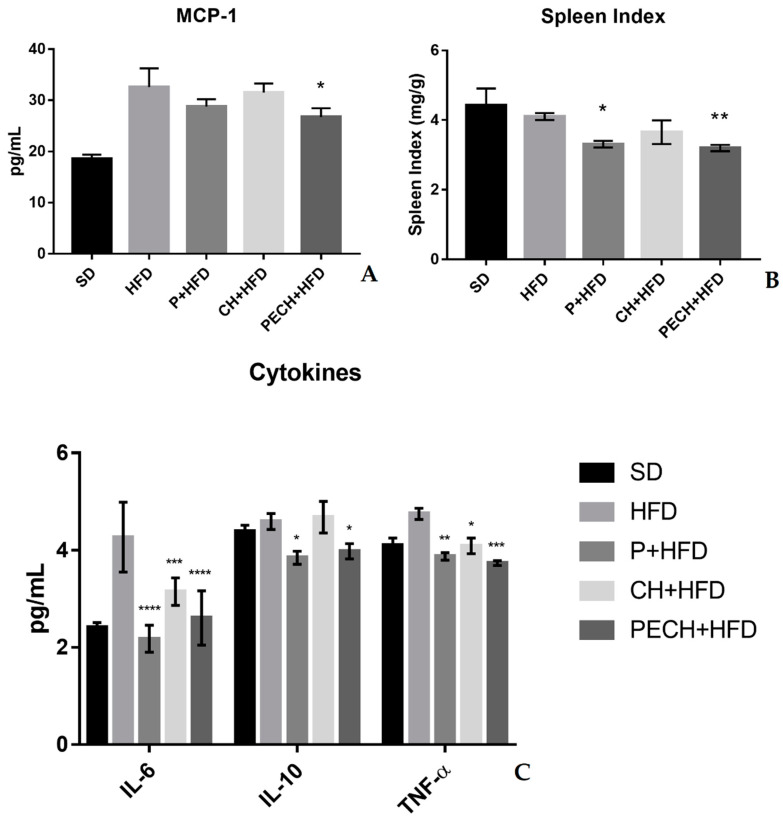
Effect of different diets on inflammatory biomarkers. (**A**) MCP-1 levels, (**B**) spleen index, (**C**) citokine levels (IL-6, IL-10, TNF-α). The different diets compared were standard diet (SD, *n* = 4), high-fat diet (HFD, *n* = 8), phenolic-enriched high-fat diet (P + HFD, *n* = 8), lyophilized cooked ham enriched high-fat diet (CH + HFD, *n* = 8) and lyophilized phenolic enriched cooked ham enriched high-fat diet (PECH + HFD, *n* = 8). HFD mean results were compared against P + HFD, CH + HFD and PECH + HFD diets, significant differences are expressed as * *p* < 0.05 ** *p* < 0.01 *** *p* < 0.001 **** *p* < 0.0001.

The concentrations of MCP-1 and cytokines are expressed in pg/mL. As represented in Figure 4, the HFD diet increased inflammatory markers compared to the SD diet. While anti-inflammatory IL-10 modulation did not show significant differences between SD and HFD, the amount of pro-inflammatory cytokines IL-6 and TNF-α and chemokine MCP-1 did it (*p* < 0.0001). 

In contrast, the consumption of the phenolics blend (P + HFD and PECH + HFD diets) decreased the concentration in plasma of cytokines IL-6, IL-10, TNF-α, and chemokine MCP-1 compared to HFD diet significantly. The global reduction of cytokines indicated lower levels of obesity-related systemic inflammation. Even the CH + HFD diet showed a lower concentration of IL-6 and TNF-α compared to HFD. In the case of the spleen index, measured as a systemic inflammatory marker, both diets, including the phenolic blend, showed significant differences (P + HFD and PECH + HFD). IL-1β was under detectable levels.

**Figure 5:**
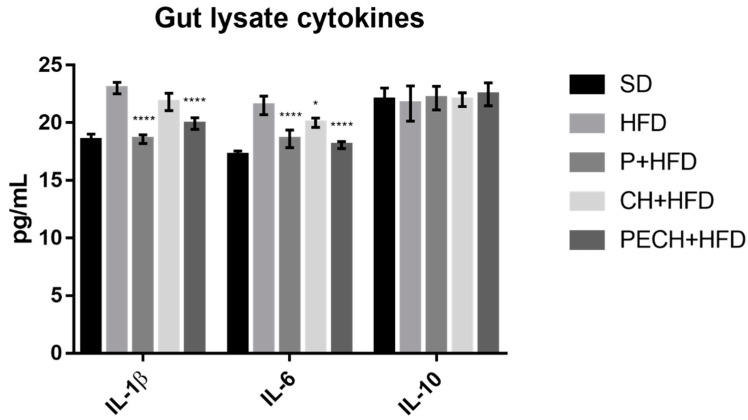
Effect of different diets on gut lysate cytokines. The different diets compared were standard diet, (SD, *n* = 4), high-fat diet (HFD, *n* = 8), phenolic-enriched high-fat diet (P + HFD, *n* = 8), lyophilized cooked ham enriched high-fat diet (CH + HFD, *n* = 8) and lyophilized phenolic enriched cooked ham enriched high-fat diet (PECH + HFD, *n* = 8) on inflammatory biomarkers IL-1β, IL-6 and IL-10 in gut samples. HFD mean results were compared against P + HFD, CH + HFD and PECH + HFD diets, significant differences are expressed as * *p* < 0.05 **** *p* < 0.0001.

Results obtained from gut lysate analysis are expressed as pg/mL (Figure 5). TNF-α and MCP-1 were under the detection limit. Statistically significant differences were detected between SD and HFD (*p* < 0.0001) in IL-1β and IL-6 levels. No statistically significant differences were observed on IL-1 between samples. Both phenolic-enriched diets, P + HFD and PECH + HFD, could have substantial effects over inflammatory status, reducing the presence of IL-1β and IL-6 on mice gut significantly. CH+HFD also had an inhibitory effect over IL-6, as shown in plasma samples.

Cytokine levels in plasma did not report differences between CH + HFD and PECH + HFD diets, except for IL-10 (*p* < 0,05). However, the consumption of a polyphenol-enriched cooked ham compared with non-enriched one showed significative differences in gut lysate, reducing both inflammatory markers IL-1β and IL-6 (*p* < 0,05).

### 3.3. Plasma Antioxidant Capacity

**Figure 6:**
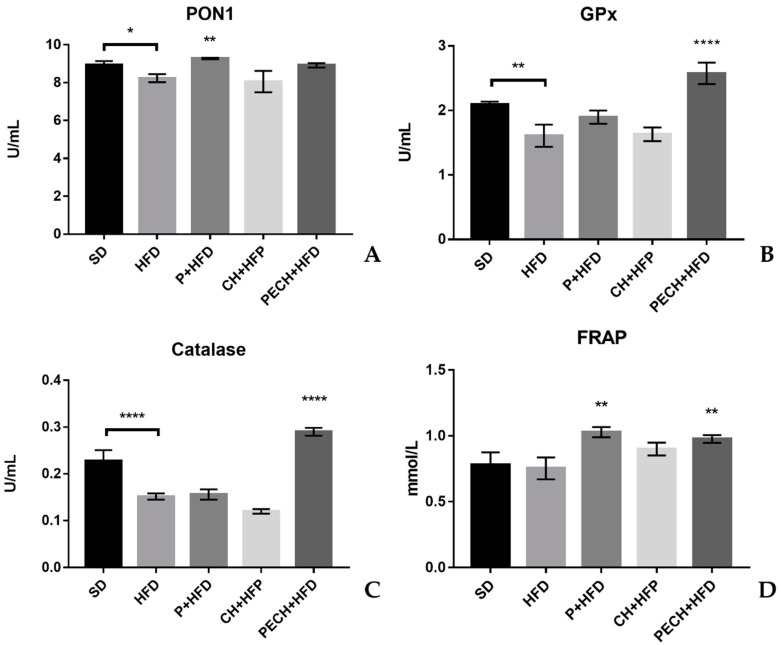
Effect of the different diets on antioxidant biomarkers. (**A**) PON1, (**B**) GPx, (**C**) Catalase, (**D**) FRAP. The different diets compared were standard diet, SD, *n* = 4), high-fat diet (HFD, *n* = 8), phenolic-enriched high-fat diet (P + HFD, *n* = 8), lyophilized cooked ham enriched high-fat diet (CH + HFD, *n* = 8) and lyophilized phenolic enriched cooked ham enriched high-fat diet (PECH + HFD, *n* = 8). HFD mean results were compared against P + HFD, CH + HFD and PECH + HFD diets, significant differences are expressed as * *p* < 0.05 ** *p* < 0.01 **** *p* < 0.0001. When they exist, differences between SD and HFD were represented with ”∩”.

Regarding plasma antioxidant capacity, the most relevant biomarkers were analysed (antioxidant enzymes were used as biomarkers of oxidative stress -PON1, GPx, Catalase- and FRAP for the overall antioxidant capacity of plasma), and represented in Figure 6 as affected for the different diets. As a general remark, the lowest levels of antioxidant enzymes indicate a poor oxidative homeostatic status. The consumption of both phenolic rich diets, P + HFD and PECH + HFD, showed increases in total antioxidant capacity of plasma measured by the FRAP method with significant differences between HFD diet (Figure 6D). Significant differences (*p* < 0.001) have been spotted in GPx and Catalase levels between SD and HFD, indicating that induced obesity can compromise antioxidant enzyme levels in plasma. 

Accordingly, a polyphenol-rich diet also resulted in an increase in activity of different antioxidant enzymes such as catalase for both P + HFD and PECH + HFD diets, PON1 with P + HFD and GPx with PECH + HFD compared to HFD. Higher levels of antioxidants enzymes imply a better response against obesity-induced oxidative stress.

### 3.4. OxLDL Results

**Figure 7:**
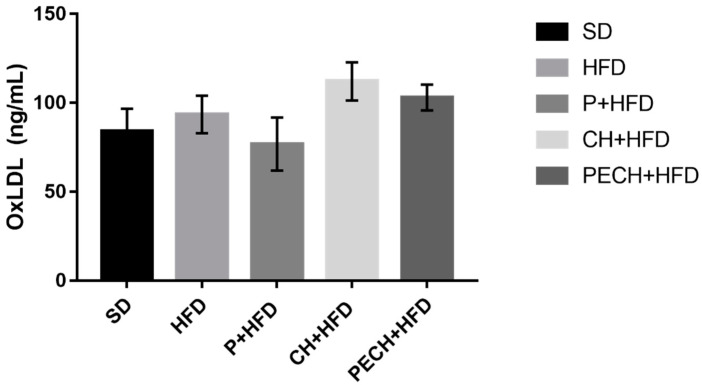
Effect of different diets on oxidized LDL (OxLDL) levels. The different diets compared were standard diet, (SD, *n* = 4), high-fat diet (HFD, *n* = 8), phenolic-enriched high-fat diet (P + HFD, *n* = 8), lyophilized cooked ham enriched high-fat diet (CH + HFD, *n* = 8) and lyophilized phenolic enriched cooked ham enriched high-fat diet (PECH + HFD, *n* = 8).

The analysis and quantification of oxLDL for each group is shown in Figure 7. Even if no significant differences were observed among groups there are two important trends in the levels of oxLDL: (1) There was a slight decrease in oxLDL levels after consumption of cooked ham (PECH + HFD) compared to the CH + HFD (that show a slight increase of oxLDL levels compared with SD) and (2) A decrease of oxLDL values after the consumption of bioactive compounds (P + HFD) compared to the SD group. 

### 3.5. Identification of Phenolic-Derived Metabolites in Urine

As expected, after the target screening strategy and the information provided by MS and MS/MS modes, six phenolic-derived metabolites were identified in urine samples from the P + HFD and PECH + HFD groups confirming the bioavailability of these phenolics (Table 2). The metabolites were detected after UPLC-QTOF analysis, although they were not quantified due to the low concentrations of the polyphenol metabolites in urine that were below their LOQ (15 nM for hydroxytyrosol glcuronide; 150 nM for 5-(3′,4′-dihydroxyphenyl-γ-valerolactone, and 4 nM for 5-(3′,4′-Dihydroxyphenyl-γ-valerolactone 3′-sulphate). No phenolic-derived metabolites were found in the rest of the groups. No phenolic-derived metabolites were detected in the remaining groups. It should be taken into account that epicatechin sulphate, 5-(3′,4′-Dihydroxyphenyl-γ-valerolactone, 5-(3′,4′-Dihydroxyphenyl)-γ-valerolactone 3′-sulphate and HXT glucuronide were detected in all urine samples from the two groups fed with the phenolic blend. However, ferulic acid 4-sulphate and feruloylquinic acid glucuronide were only identified in the group fed with the enriched phenolic compounds diet alone (P + HFD).

## 4. Discussion

One of the main drawbacks to link the beneficial health effects of phenolics with the dietary phenolics intake is the poor bioavailability of these compounds. Besides, most of these phenolics are extensively metabolized by the gut microbiota to other derived metabolites that present higher absorption. Next, they undergo extensive phase-II metabolism yielding, mainly glucuronide and sulphate conjugates that can persist in systemic circulation up to 3–4 days after intake [37,38]. In our study, the identification of six phenolic-derived metabolite conjugates (Table 2) in the urine of mice fed with phenolic-enriched ham confirmed the bioavailability of phenolics and suggested that the effects observed could be mediated by their action. 

Bodyweight changes were evaluated to prove the effectivity of high-fat diets, which were supposed to increase pathogenicity related biomarkers [7]. Changes complied with diet specification sheet and with previous studies [39,40]. Non statistically significant differences were found between diet-induced obesity groups regarding body weight. Body composition changes had corroborated higher adipose tissue in the high-fat diet groups. Adipose tissue directly correlated with the presence of inflammatory biomarkers such as IL-6 and TNF-α [41,42]. Variation in lipid infiltration in liver also showed the effectiveness of high-fat diets inducing obesity [43]

The spleen initiates the immune reaction to blood-borne antigens [44]. The spleen index reflects the prognosis of diseases and changes in this index respond to the nonspecific immunity of the organism [45]. Splenomegaly, which mainly occurs during active diseases [46], can be used as an inflammatory marker due to its increase in pro-inflammatory situations such as metabolic syndrome [47]. In the present study, the spleen index has shown significant differences between both polyphenol-enriched diets (P + HFD, PECH + HFD) and non-treated obese group.

Cytokines, measured as inflammatory mediators [48], have shown similar trends in inflammatory modulation. On the other hand, non-significant changes were observed in oxLDL levels. Cytokine IL-6 can be both inflammatory and anti-inflammatory, depending on certain conditions. Through metabolic syndrome conditions, higher levels of IL-6 have been linked with cardiovascular diseases, being people with the highest level of IL-6 twice to five times more likely to have a heart attack, stroke, or another cardiovascular episode [49,50]. In this study, all three treatment diets have shown a modulatory effect over IL-6 with statistically significant differences for the high-fat diet. While phenolic-enriched treatments showed the lowest IL-6 levels, especially in the gut, even the cooked ham enriched diet reduced its concentration. Those effects were previously corroborated on an *in-vitro* cell assay [51] and in mice gut [52]. While TNF-α is an inflammatory cytokine, which is downregulated through phenolic-enriched diets [53,54], IL-10 is a pleiotropic anti-inflammatory cytokine [55]. Its depletion, which was not as pronounced as those of TNF-α or IL-6, could be justified through the reduction of its expression by the treatments, but further studies, which should include PCR analysis, should be performed confirm these results. Chemokine MCP-1 is involved in macrophage recruitment. Local proliferation of macrophages has been shown to contribute to obesity-associated adipose tissue inflammation [56]. However, another study suggested that MCP-1 might be a necessary component of the inflammatory response for adipose tissue protection [57]. In this study, non-significant differences were observed through intervention in most of the cases, despite of PECH + HFD, which showed a slight, but significant reduction. The reduction of MCP-1 can result in a reduction of cardiovascular disease risk [58]. IL-1β is a pro-inflammatory cytokine produced by macrophages in response to TNF-α. Previous studies in mice gut have corroborated that polyphenols can have an inhibitory effect, resulting in a downregulation of inflammatory processes [59,60,61]

PON1 is an antioxidant enzyme synthesized primarily in the liver, and it is associated with high-density lipoproteins in serum [62]. Its role is to degrade toxic organophosphates and metabolize oxidized lipids [63], and lower levels of PON1 have been correlated with the metabolic disease [64]. Phenolic compounds have been proved to raise PON1 levels due to an aryl hydrocarbon receptor-dependent mechanism [65], and previous studies have demonstrated that polyphenol-enriched food increased PON1 levels [66,67]. While P + HFD has been shown to raise PON1 levels, no significant differences were achieved through PECH + HFD, which can be explained by the lower concentration of phenolic compounds. GPx is one of the main antioxidant enzymes, and its depletion is linked with the increase of free radicals [68]. Polyphenol supplementation raised GPx levels, preventing from environmental injuries [69]. Higher levels of GPx have been associated with the prevention of some diseases such as cancer and cardiovascular disease [70]. FRAP results imply a measure of overall antioxidant capacity in plasma. Higher levels of phenolic compounds have been correlated with higher antioxidant capacity, which protects against previously mentioned diseases through neutralization of reactive oxygen species [71,72,73,74]. No differences were found between SD and HFD diet for the FRAP assay characteristics. FRAP detects free reducing compounds levels, and those are not supposed to change between a standard diet and a non-antioxidant-enriched high fat diet. Differences between HFD and CH + HFD could be explained by the antioxidants present in processed meat, such as vitamin C. Catalase is another antioxidant enzyme involved in the protection against oxidative damage through reactive oxygen species. Some studies have suggested that catalase plasma concentration can be reduced using some antioxidants like Vitamin E [75], but, in the present study, statistically significant differences were found between polyphenol enriched diets and high-fat diet. Catalase deficiency can worsen metabolic syndrome and lead to neurological and other disorders [76]. Therefore, the increase of catalase concentration in plasma, as observed in the present study, may have protective effects against age-related oxidative stress [77].

The differences observed between P + HFD and PECH + HFD on antioxidant enzymes modulation were also found in previous studies [44] and suggest that bringing the phenolic blend through processed food could have some effects due to processing (i.e., thermal processing, additives). Another difference between P + HFD and PECH + HFD is the presence of different peptides from cooked ham, which can also exert antioxidants effect [78]. Studying the mentioned factors could lead to exciting research about processed foods and polyphenol enrichment. To sum up, both P + HFD and PECH + HFD have shown significant modulation of inflammatory and oxidative status, changes which could lead to the prevention of metabolic syndrome derived diseases such as cardiovascular diseases and cancer.

## 5. Conclusions

Results in the murine induced obesity model, demonstrate a robust statistically significant improvement in key parameters related with obesity risk in the groups feed with a phenolic-enriched diet (P + HFD and PECH + HFD). In both groups there was an improvement in body composition parameters (Table 3), inflammatory biomarkers and antioxidant enzymes levels. Specifically, in the group feed with the phenolic enriched cooked ham (PECH + HFD) there was an improvement of total Fat volume, spleen index, plasmatic MCP1, IL-6, Il-10, TNF-α, gut IL-1β, gut IL-6 and GPx and catalase enzymes.

Consuming foods rich in antioxidant compounds could be a new dietary strategy to mitigate the chronic inflammation and reducing oxidative stress associated with overweight or obesity and subsequent onset of other chronic non-communicable diseases. The dietary intake of some phenolic compounds, present only in certain plant foods, have shown significant antioxidant and anti-inflammatory activity through various possible mechanisms in different biological systems. Although some meat products also contain certain biopeptides with antioxidant activity, in addition to other essential nutrients, very little research has focused on studying the potential beneficial effects of consuming phenolic compounds in combination with processed meat products. The main findings of this study suggested that the consumption of a low fat and salt cooked ham and enriched with a mixture of dietary phenolic compounds exert a protective effect against the inflammatory status and the oxidative stress in a pre-clinical study of diet-induced obesity in C57/BL6J mice. These results suggest that the functionalized cooked ham with the appropriate dietary phenolics could result in the potential decrease of the risk of certain non-communicable chronic diseases. Those promising results should be further studied through human clinical assays to corroborate these potential health benefits.

## Figures and Tables

**Table 1:** Macronutrient profile of standard diet and high-fat diets used throughout the study.

Macronutrients Composition and Energy Density	Standard Diet	High-Fat Diets
	(SD)	(HFD)
Protein (%)	14.3	23.5
Total carbohydrate (%)	48.0	27.3
Fiber (%)	4.1	6.5
Fat (%)	4.0	34.3
Energy density (Kcal/g)	2.9	5.1
• Calories from protein (%)	20	18.3
• Calories from carbohydrates (%)	67	21.4
• Calories from fat (%)	13	60.3

**Table 2:** Phenolic derived metabolites identified in urine.

Metabolites	RT (min)	Experimental *m/z*	MS/MS Fragments	Occurrence	
				P + HFD	PECH + HFD
Epicatechin sulphate	5.2	369.0286	125.0235; 109.0289; 289.0708	+	+
5-(3′,4′-Dihydroxyphenyl-γ-valerolactone	5.4	207.0663	163.0764; 122.0369	+	+
5-(3′,4′-Dihydroxyphenyl-γ-valerolactone 3′-sulphate	3.9; 5.3	303.0180	223.0605; 179.0702	+	+
Ferulic acid 4-sulphate	8.3	273.0074	193.0505; 134.0379	+	−
Feruoylquinic acid glucuronide	5.5	543.1355	367.1013; 498.9785; 173.0450	+	−
Hydroxytyrosol glucuronide	3.0	329.0878	123.0445; 153.0169; 176.0381; 131.0349	+	+

Analytical parameters for the determination of phenolic-derived metabolites in urine samples using UPLC-ESI-QTOF-MS. The different diets compared were phenolic-enriched high-fat diet (P+HFD) and lyophilized phenolic enriched cooked ham enriched high-fat diet (PECH+HFD, *n* = 8).

**Table 3:** Summary of comparisons between high fat diet group (HFD) and both phenolic enriched high fat diet (P+HFD) and phenolic enriched cooked ham high fat diet (PECH + HFD).

Oxidative and Inflammatory Markers		Does it Improve Compared to HFD Group in…	
		P + HFD?	PECH + HFD?
Physiological parameters	Bodyweight	NO	NO
	Adipose tissue volume	YES ***	YES **
	Hepatic fat	YES ***	NO
	Spleen Index	YES *	YES **
Inflammatory markers (plasma)	MCP-1	NO	YES *
	IL-6	YES ****	YES ***
	IL-10	YES *	YES *
	TNF-α	YES **	YES ***
Inflammatory markers (gut)	IL-1β	YES ****	YES ****
	IL-6	YES ****	YES ****
	IL-10	NO	NO
Oxidative biomarkers	PON1	YES**	NO
	GPX	NO	YES ****
	CATALASA	YES *	YES ****
	FRAP	YES **	YES **
	PON1	NO	NO

Statistically significant differences are expressed as * *p* < 0.05 ** *p* < 0.01 *** *p* < 0.001 **** *p* < 0.0001.

## Supplementary Materials

The following are available online at https://www.mdpi.com/2076-3921/9/7/639/s1, Table S1: Phenolic compounds and derived metabolites searched in the urine samples by UPLC-ESI-QTOF-MS.
