# Supplementary material for: Anti-Inflammatory and Antioxidant Effects of Regular Consumption of Cooked Ham Enriched with Dietary Phenolics in Diet-Induced Obese Mice
Source: Antioxidants (Basel). 2020 Jul 21;9(7):639. doi: 10.3390/antiox9070639 (PMC7402095; doi:10.3390/antiox9070639)
Supplement: Supplementary file 1 [file antioxidants-09-00639-s001.pdf]

Table S1. Phenolic compounds and derived metabolites searched in the urine samples by UPLC-ESI-QTOF-MS.

Hydroxy-tyrosol (HXT)	Homovanillic acid/alcohol sulphate	3-O-Feruloylquinic acid	EC-O-Glucuronide	4′-O-Methyl-EGC-O-sulphate	5-(3′,4′/3′,5′/4′,5′)-DHP)- γ-VL glucuronide sulphate
Methyl-HXT	Caffeic acid	Feruoylquinic acid glucuronide	EC-O-Sulphate	4′-O-Methyl-EGC-O-sulfoglucuronide	5-(3′,4′/3′,5′/4′,5′)-DHP)- γ-VL glucuronide disulphate
HXT-glucuronide	3-O-Caffeoyl-quinic acid (chlorogenic acid)	Dihydroferulic acid	EC-O-Sulfoglucuronide	(Epi)Catechin gallate *(ECG)*	O-Methyl- 5-(3′,4′/3′,5′/4′,5′)-DHP)- γ-VL glucuronide
HXT-sulphate	Caffeic acid 3/4-sulphate	Dihydroferulic acid 4-O-glucuronide	(3′/4′)-methyl-ec	Gallic acid	O-Methyl- 5-(3′,4′/3′,5′/4′,5′)-DHP)- γ-VL sulphate
HXT-methyl ether	Dihydrocaffeic acid 3-O-glucuronide	Dihydroferulic acid 4-sulphate	(3′/4′)-Methyl-EC-O-sulphate	(3′/4′)-O-Methylgallic acid	5-(3′,4′,5′-THP)-γ- VL
HXT-methyl ether glucoside	Dihydrocaffeic acid 3-sulphate	*p*-Coumaric acid	(Epi)gallocatechin (*egc*)	HP Valeric acid glucuronide	5-(3′,4′,5′-THP)-γ- VL glucuronide
Tyrosol (ty)	Caffeic acid 3/4-O-glucuronide	*p*-Coumaric acid sulphate	Egc-o-glucuronide	5-(HP)-γ-VL glucuronide	5-(3′,4′,5′-THP)-γ- VL sulphate
Ty-glucuronide	(Iso)Ferulic acid	4-Ethylcatechol	EGC-O-sulphate	5-(HP)-γ-VL glucuronide sulphate	5-(3′,4′,5′-THP)-γ-VL glucuronide sulphate
Ty-sulphate	Ferulic acid 4-O-sulphate	Ethylcatechol glucuronide	(3′/4′)-O-Methyl-EGC	5-(3′,4′/3′,5′)-DHP)- γ-VL	Methyl-5-(3′,4′,5′-THP)-γ-VL- glucuronide
Homovanillic acid/alcohol	Ferulic acid 4-O-glucuronide	(Epi)catechin *(ec)*	4′-O-Methyl-EGC-O-glucuronide	5-(3′,4′/3′,5′/4′,5′)-DHP)-γ-VL glucuronide	Methyl-5-(3′,4′,5′- THP)-γ-VL sulphate
